# Novel immortal human cell lines reveal subpopulations in the nucleus pulposus

**DOI:** 10.1186/ar4597

**Published:** 2014-06-27

**Authors:** Guus GH van den Akker, Don AM Surtel, Andy Cremers, Ricardo Rodrigues-Pinto, Stephen M Richardson, Judith A Hoyland, Lodewijk W van Rhijn, Tim JM Welting, Jan Willem Voncken

**Affiliations:** 1 Department of Orthopaedic Surgery, Maastricht University Medical Centre, Maastricht Postbox 616, 6200 MD, the Netherlands; 2 Department of Molecular Genetics, Maastricht University Medical Centre, Maastricht, the Netherlands; 3 Centre for Regenerative Medicine, Institute of Inflammation and Repair, University of Manchester, Oxford Road, Manchester M13 9PL, UK; 4 Department of Orthopaedics, Central Hospital of Porto–St Anthony Hospital, Porto, Portugal

## Abstract

**Introduction:**

Relatively little is known about cellular subpopulations in the mature nucleus pulposus (NP). Detailed understanding of the ontogenetic, cellular and molecular characteristics of functional intervertebral disc (IVD) cell populations is pivotal to the successful development of cell replacement therapies and IVD regeneration. In this study, we aimed to investigate whether phenotypically distinct clonal cell lines representing different subpopulations in the human NP could be generated using immortalization strategies.

**Methods:**

Nondegenerate healthy disc material (age range, 8 to 15 years) was obtained as surplus surgical material. Early passage NP monolayer cell cultures were initially characterized using a recently established NP marker set. NP cells were immortalized by simian virus 40 large T antigen (SV40LTag) and human telomerase reverse transcriptase expression. Immortalized cells were clonally expanded and characterized based on collagen type I, collagen type II, α1 (COL2A1), and SRY-box 9 (SOX9) protein expression profiles, as well as on expression of a subset of established *in vivo* NP cell lineage markers.

**Results:**

A total of 54 immortal clones were generated. Profiling of a set of novel NP markers (*CD24*, *CA12*, *PAX1*, *PTN*, *FOXF1* and *KRT19* mRNA) in a representative set of subclones substantiated successful immortalization of multiple cellular subpopulations from primary isolates and confirmed their NP origin and/or phenotype. We were able to identify two predominant clonal NP subtypes based on their morphological characteristics and their ability to induce SOX9 and COL2A1 under conventional differentiation conditions. In addition, cluster of differentiation 24 (CD24)–negative NP responder clones formed spheroid structures in various culture systems, suggesting the preservation of a more immature phenotype compared to CD24-positive nonresponder clones.

**Conclusions:**

Here we report the generation of clonal NP cell lines from nondegenerate human IVD tissue and present a detailed characterization of NP cellular subpopulations. Differential cell surface marker expression and divergent responses to differentiation conditions suggest that the NP subtypes may correspond to distinct maturation stages and represent distinct NP cell subpopulations. Hence, we provide evidence that the immortalization strategy that we applied is capable of detecting cell heterogeneity in the NP. Our cell lines yield novel insights into NP biology and provide promising new tools for studies of IVD development, cell function and disease.

## Introduction

Degenerative disc disease (DDD) poses a substantial socioeconomic burden in developed countries [1]. Currently, treatment of DDD is primarily aimed at relieving symptoms because effective therapy to delay or prevent DDD is not available.

The intervertebral disc (IVD) consists of a central gelatinous nucleus pulposus (NP) encircled by an elastic, ligamentous annulus fibrosus (AF) and is flanked superiorly and inferiorly by cartilaginous endplates. NP cells are highly specialized and share some features with articular chondrocytes in terms of aggrecan (ACAN), collagen type II, α1 (COL2A1), and SRY-box 9 (SOX9) protein expression [2]. However, compared to articular cartilage (AC), the NP maintains a unique extracellular matrix (ECM) with a higher glycosaminoglycan to hydroxyproline (GAG/OH-pro) ratio, and its native cells display distinctive gene expression signatures [3-5]. The initial stages of DDD correlate with reduced cellularity, aberrant cell function, loss of proteoglycans and concomitant tissue dehydration [6]. As cells within the IVD are responsible for ECM maintenance and homeostasis, they play an important role in the degenerative process. The findings in an increasing number of studies support the idea that mature NP cells are derived from precursor notochordal cells (NCs), although NP cells differ from NCs morphologically and express different genes (reviewed in [7]). However, it is becoming increasingly clear that the NP comprises multiple cell subpopulations [8-11]. This cellular heterogeneity may reflect different stages of proliferation, differentiation and maturation; however, relatively little is known about these NP cell subpopulations. Successful development of cell replacement therapies and IVD regeneration is crucially dependent on an in-depth understanding of cellular and molecular characteristics of the functional IVD. To accomplish this, access to representative human cell models is pivotal. However, current research on primary cells is hampered by restricted availability of human cells, particularly from nondegenerate discs, where there is a relatively inherent low cellularity within the tissue. In addition, lack of well-defined cellular characteristics and differences in the origin of study material (for example, donor age, IVD degeneration status) underlies experimental variability and thus low reproducibility. To date, a few NP cell lines have been independently generated by Sakai *et al*. [12] and, more recently, by Liu and co-workers [13].

As no cell lines are available that represent the reported different subpopulations in adult human NP cells, we set out to generate *in vitro* cell models for human NP cells. Our approach using immortalization, clonal selection and outgrowth allowed us to address NP cell heterogeneity. Here we provide a molecular and cellular characterization of the first immortal human NP cellular subpopulations.

## Methods

### Isolation of intervertebral disc cells, cell culture and immortalization

Nondegenerate healthy disc material was obtained as surplus material from correction surgery (Maastricht University Medical Centre Medical Ethical Review Committee (MERC) approval 08-4-028). Under Dutch law, informed patient consent is part of the MERC approval and is not required separately (see Table 1). Determination of the absence of DDD was based on macroscopic examination. Tissue samples were macroscopically dissected by the staff surgeon. Only IVD tissue from the convex side of the scoliotic disc was used for processing. To prevent cross-contamination between IVD-derived tissues, remnant endplate material was completely resected and a broad section of the transition zone extending well into defined NP tissue or outer AF tissue was removed. NP and AF tissues were stored and processed separately. Tissue was dissected into small pieces and digested overnight with 0.05% collagenase type II (17101-015; Invitrogen, Carlsbad, CA, USA) in Dulbecco’s modified Eagle’s medium/Nutrient Mixture F-12 (GlutaMAX DMEM/F-12; Gibco, Grand Island, NY, USA) buffered with 4-(2-hydroxyethyl)-1-piperazineethanesulfonic acid at 37°C under constant agitation. Isolates were passed through a 70-μm cell strainer and collected by centrifugation (280 × *g*, 5 minutes, Eppendorf centrifuge 5810R) and cultured in maintenance medium (Mmed) (DMEM-F-12/GlutaMAX, 10% foetal calf serum (DE14-801 F; BioWhittaker, Walkersville, MD, USA), 1% penicillin-streptomycin (Gibco) and 1% nonessential amino acids (NEAAs) (Gibco). Cells were seeded at a density of 30,000 cells/cm^2^. Upon confluence (passage 0 (P0)), cultures were expanded as pools (1:2 dilutions per passage, until P5) to obtain sufficient material for initial characterization. Monolayer differentiation was induced using an established protocol for articular chondrocytes [14]. A total of 30,000 cells/cm^2^ were incubated in differentiation medium (Dmed: DMEM/F-12, 1% antibiotic/antimycotic, 1% insulin/transferrin/sodium selenite (ITS; Invitrogen)), 1% L-ascorbic acid 2-phosphate deoxycholate (Sigma-Aldrich, St Louis, MO, USA), 1 ng/ml transforming growth factor β3 (TGFβ3) (PHG9305; Gibco) and 1% NEAA for the indicated time periods. The U-CH1 chordoma cell line (courtesy of S Brüderlein, Ulm, Germany) was grown as previously described [15].

**Table 1:** **Intervertebral disc donor characteristics and clones**^**a**^

							**Morphology**		
**Donor**	**Sex**	**Age**^**b**^**, yr**	**Medical indication**	**Position IVD**	***T*** **mRNA primary isolates**	**NP clones**	**Wave**	**Cobbl**	**Tiny**
D1	M	8	Spina bifida/scoliosis	T11-L4	Positive	N/A			
D2	F	13	Idiopathic scoliosis	L1-L4	Negative	N/A			
D3	M	14	Spina bifida/scoliosis	T12-L4	Negative	N/A			
D4	F	15	Idiopathic scoliosis	T7-T10	Negative	34	19	14	1
D5	M	13	Spina bifida/scoliosis	T6-L1	Negative	20	11	7	2

^a^IVD, Intervertebral disc; L, Lumbar; N/A, Not applicable; NP, Nucleus pulposus; T, Thoracic. ^b^Age in years at the time discs were obtained during surgery. Tissues were obtained from young adolescent scoliosis patients who had undergone correction surgery. In contrast to herniated or adult discs, these intervertebral discs showed no signs of degeneration; they had clear, lucid nuclei pulposi that could easily be distinguished from the annulus fibrosis. Each cell isolate (from donors D1 to D5) derived from AF and NP tissue excised at multiple adjacent levels, ranging from T6 to L4 as indicated, in a single individual. The total number of generated clones is indicated in column ‘Immortal NP clones’, and the amount of cobblestone (Cobbl), wavelike (Wave) and tiny clones is indicated in column ‘Clonal morphology’. Brachyury T (*T*) expression was measured in all primary isolates at the mRNA and protein levels; only D1 was found to be positive.

### Retroviral transduction and generation of immortal clones

The simian virus 40 large T antigen (SV40LTag) and human telomerase reverse transcriptase (hTERT) cDNAs were cloned into pBABE-hygro [16] and pBMN-IRES-NEO vectors, respectively. Production of viral particles was performed as described previously [17]. Viral titres were sufficiently high to achieve nearly 100% infection. Retrovirally transduced P5 AF and NP cells from donors 4 and 5 were selected with 400 μg/ml G418 and 25 μg/ml hygromycin B (PAA Laboratories/GE Healthcare Life Sciences, Somerset, UK). Immediately following selection, clones were generated by plating less than 1 cell (that is, ±0.5) per well in 96-well plates in reduced selection medium (200 μg/ml G418 and 12 μg/ml hygromycin B). After 1 week of culture, small colonies became discernible. Culture wells with more than one colony were omitted from further study to ensure single picks. Single clones were expanded under continued antibiotic selection pressure and expanded for three more passages (P1 to P4) for cryogenic storage and screening experiments. Selected clones were expanded and remained stable for 8 months or 328 additional population doublings.

### Telomeric repeat amplification protocol

To measure TERT activity, a telomeric repeat amplification protocol assay was performed according to the manufacturer’s instructions (TRAPeze Telomerase Detection Kit; Millipore, Amsterdam, the Netherlands) [18]. Briefly, 1 μg of total protein input was used to produce tandem TAAGGG repeats (extension step at 30°C for 30 minutes); these repeats were amplified by PCR using telomere-specific primers (30 cycles), which typically generated a ladder of products with 6-bp increments starting at 50 nucleotides (for example, 50, 56, 62, 68).

### Aggrecan coating differentiation assays

To assess NP clonal responses to a relevant proteoglycan in the NP ECM, ACAN was used to coat culture dishes. In brief, we added 5 μg of purified bovine ACAN (Sigma-Aldrich A1960) in 200 μl of solution to 24-well culture plates. The continuously agitated solution was allowed to evaporate overnight at ambient temperature under sterile conditions as described previously [19]. Plates were washed twice with phosphate-buffered saline (PBS), and 200,000 cells/well suspended in Dmed were seeded. Media were changed every other day.

### Matrigel cell culture assays

To assess increases in NP clonal behaviour in a three-dimensional environment, we established a Matrigel hydrogel system to support chondrogenesis of articular chondrocytes. Briefly, 90 μl of growth factor–reduced Matrigel (BD Biosciences, San Jose, CA, USA) was dispensed into 12-well culture plates (Greiner Bio-One, Monroe, NC, USA). NP cells (*N* = 68,400) were grown for 7 days under conventional (that is, articular chondrocyte) differentiation conditions (in Dmed). mRNA samples were taken at 7 days and isolated using the mirVana mRNA Isolation Kit (Ambion, Austin, TX, USA) for whole RNA extraction according to the manufacturer’s instructions.

### RNA isolation and quantitative real-time PCR

For RNA isolation, cells were disrupted in TRIzol reagent (Invitrogen). RNA isolation, RNA quantification (UV) spectrometry (Nanodrop, Thermo Scientific) and cDNA synthesis were performed as described before [20]. Real-time quantitative PCR (RT-qPCR) was performed using Mesagreen qPCR MasterMix Plus for SYBR Green (Eurogentec). Validated primer sets used are depicted in Table 2. An Applied Biosystems ABI PRISM 7700 Sequence Detection System was used for amplification achieved by initial denaturation 95°C for 10 minutes followed by 40 cycles of DNA amplification. Data were analysed using the standard curve method and normalized to β-actin (bACT).

**Table 2:** **Quantitative PCR primer list**^**a**^

**Symbol**	**Gene name**	**Tissue**	**Forward primer**	**Reverse primer**
*ACAN*	Aggrecan	AC, NP	GCAGCTGGGCGTTGTCA	TGAGTACAGGAGGCTTGAGGACT
*CA12*	Carbonic anhydrase XII	NP	ATCCAACTAATGCCACCACCAA	TGAGACCACGAAGAGACTGGCT
*COL1A1*	Collagen type I, collagen α1	AF	TGGAGAGTACTGGATTGACCCC	TGCAGAAGACTTTGATGGCATC
*COL2A1*	Collagen type II, collagen α1	AC, NP	TGGGTGTTCTATTTATTTATTGTCTTCCT	GCGTTGGACTCACACCAGTTAGT
*COMP*	Cartilage oligomeric matrix protein	AC	CAAGGCCAACAAGCAGGTTTG	CAGTTATGTTGCCCGGTCTCA
*CD24*	Cluster of differentiation 24	NP	CCACGCAGATTTATTCCAGTGA	GCCAACCCAGAGTTGGAAGTAC
*FOXF1*	Forkhead box F1	NP	CCCACACAGGAATTCTGCTGA	TTCCCCCACTTCTGCCATT
*KRT19*	Keratin 19	NC, NP	GCAGTCACAGCTGAGCATGAA	TCCGTTTCTGCCAGTGTGTCT
*PAX1*	Paired box 1	NP	AGAGCCTGACATCGCCTGTTAA	CGCTTTCCTTTATTCAGAGGCA
*PTN*	Pleiotrophin	NP	AGAAGCAATTTGGCGCGGA	TTCAGGTCACATTCTCCCCAGG
*SOX9*	SRY-box 9	AC, NP	AGTACCCGCACCTGCACAAC	CGCTTCTCGCTCTCGTTCAG
*T*	Brachyury T	NC	CCACCTGCAAATCCTCATCCT	TTGGAGAATTGTTCCGATGAGC

^a^AC, Articular cartilage; AF: Annulus fibrosus; NC, Notochordal cell; NP, Nucleus pulposus. The sequences are the validated real-time primer sets used for the indicated genes that were subjected to quantitative PCR analysis. ‘Tissue’ column lists tissue in which marker expression is reported.

### Protein extraction and immunoblotting

Protein extraction and immunoblotting were performed and analysed as described previously with minor adjustments [21]. For extraction, cells were lysed in radioimmunoprecipitation assay buffer (50 mM Tris, pH 8.0, 150 mM NaCl, 0.1% SDS, 5 mM ethylenediaminetetraacetic acid (EDTA), 0.5% w/v sodium deoxycholate and 1% Nonidet P-40) supplemented with protease and phosphatase inhibitors (Roche Diagnostics, Indianapolis, IN, USA). Lysates were sonicated on ice using the Soniprep 150 Plus ultrasonic disintegrator (MSE, London, UK) at amplitude 10 for 14 cycles (1 second on, 1 second off). Insoluble material was removed by centrifugation (10 minutes at 16,000 × *g* and 4°C). Protein concentration was determined using a bicinchoninic acid protein assay kit (Pierce/Thermo Scientific, Rockford, IL, USA). Protein samples were separated by SDS-PAGE and immobilized on nitrocellulose membranes. Membranes were blocked for 1 hour in 5% nonfat dry milk powder (FrieslandCampina, Amersfoort, the Netherlands) at ambient temperature and then incubated with primary antibodies overnight at 4°C. Antisera used were polyclonal goat anti-COL2A1 (1320-01; Southern Biotech, Birmingham, AL, USA), polyclonal goat collagen type I, α1 (anti-COL1A1) (1310-01; SouthernBiotech), polyclonal goat anti-SOX9 (ab3697; Abcam, Cambridge, UK), rabbit polyclonal antibody against brachyury T (hereafter ‘T’) (H-210 and SC-20109; Santa Cruz Biotechnology, Santa Cruz, CA, USA), mouse monoclonal anti-SV40LTag (Pab 108, sc-148; Santa Cruz Biotechnology), rabbit polyclonal anti-TERT (R1187; Acris Antibodies, San Diego, CA, USA), mouse monoclonal β-actin (clone C4, 08691001; MP Biomedicals, Santa Ana, CA, USA), mouse monoclonal α-tubulin (clone B-5-1-2, T6074; Sigma-Aldrich), rabbit polyclonal KAP-1 (A300-275A; Bethyl Laboratories, Montgomery, TX, USA). Secondary antisera used were polyclonal rabbit anti-goat (P0449; Dako Cytomation, Glostrup, Denmark), rabbit anti-mouse (P0260; Dako) and donkey anti-rabbit (711-035-152; Jackson ImmunoResearch Laboratories, West Grove, PA, USA). Signals were detected using enhanced chemiluminescence (Pierce/Thermo Scientific).

### Flow cytometry

Flow cytometry was performed as described previously [22]. Cell surface markers analysed were cluster of differentiation 44 (CD44), CD73, CD90 and CD105 (Miltenyi Biotec, San Diego, CA, USA); gangliosidase 2 (GD2) (Santa Cruz Biotechnology); and CD24 (BioLegend, San Diego, CA, USA). Briefly, antibody incubation (10 minutes at 4°C in the dark) was followed by washes.

### Cell proliferation assays

Cells were seeded at 6,400 cells/cm^2^, allowed to attach overnight (*t*0; baseline) and grown in 12-well plates for 12 days. At the indicated time points (*cf*. Figure 2C), cells were washed in PBS, fixed in 3.7% formaldehyde in PBS solution for 10 minutes at ambient temperature and rinsed with demineralized water. Nuclear DNA was stained (0.1% Crystal violet, 30 minutes, ambient temperature), after which cells were washed extensively with demineralized water. Crystal violet was extracted at a fixed volume of 10% acetic acid. Absorbance was determined at 590 nm (Benchmark microplate reader; Bio-Rad Laboratories, Hercules, CA, USA).

### Biochemical matrix assays

Total sulphated GAG content was determined in papain-digested samples by using 1,9-dimethylmethylene blue stain (DMB) (Polysciences, Eppelheim, Germany) [23]. Briefly, cell cultures were washed with 0.9% NaCl and incubated at 60°C for 16 hours in digestion buffer (100 mM sodium phosphate, pH 6.5, 5 mM L-cysteine HCl, 5 mM EDTA) containing 125 to 140 μg/ml papain (Sigma-Aldrich P3125). Samples were collected and centrifuged at 12,000 rpm for 5 minutes, and the supernatants were incubated with DMB solution (46 μM DMB, 40.5 mM glycine, 40.5 mM NaCl, pH 1.5). An absorption ratio of 540 nm/595 nm was determined within 5 to 10 minutes of adding DMB solution. A standard curve of chondroitin sulphate (Sigma-Aldrich) was included as a reference. OH-pro quantification was performed using a chloramine-T assay and compared to a *trans*-4-hydroxyproline standard essentially as described previously [24]. Both GAG and OH-pro content were normalized against cellular DNA content. DNA concentration in papain-digested samples was determined using SYBR Green stain (Eurogentec, Fremont, CA, USA) as described previously [14]. Quantification of DNA concentration was analysed against a genomic control DNA standard (calf thymus; Invitrogen).

### Statistics

Statistical significance (*P* < 0.05) was determined by two-tailed Student’s *t*-test. To test for normal distribution of input data, D’Agostino–Pearson omnibus normality tests were performed. All quantitative data sets presented passed the normality tests. The results of gene expression analyses are reported as means with standard deviations where indicated. The *P*-values given in the figure legends are for comparisons between AF and NP for all donors combined or between clonal subtypes (NP-R vs NP-nR). *P*-values for every statistical calculation are presented in Additional file 1: Tables S1 to S4.

## Results

### Phenotypic characterization of primary nucleus pulposus cultures

**Figure 1:**
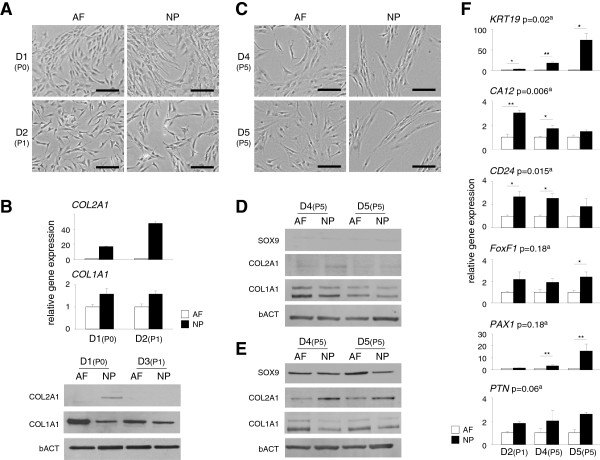
**Confirmation of the nucleus pulposus cell phenotype *in vitro. *(A)** Representative phase-contrast images of primary nucleus pulposus (NP) and annular fibrosus (AF) cells from donor 1 (D1) and donor 2 (D2). Passage numbers (P*x*) for each donor’s tissue are indicated in parentheses (for example, D1 (P0)). Black bars = 20 μm. **(B)***Top*: Graphed results of gene expression analysis of the chondrocyte markers collagen type I, α1 (*COL1A1*), and collagen type II, α1 (*COL2A1*), in primary AF (white bars) and NP (black bars) cell isolates from D1 (P0) and D2 (P1) tissues, respectively. Gene expression was normalized to β-actin (bACT) mRNA levels. Data presented are relative to the AF values. *Lower panels*: Immunoblot analysis of primary AF and NP cell lysates from two independent donors, D1 (P0) and D3 (P1), respectively, for COL1A1 and COL2A1. bACT was used as a loading control. **(C)** Representative phase-contrast images of AF and NP cultures from D4 and D5, both at P5. Black bars = 20 μm. **(D)** Immunoblot analysis of COL1A1, COL2A1 and SRY-box 9 (SOX9) on cell lysates from P5 AF and NP cells. **(E)** P5 AF and NP cells were stimulated for 7 days with differentiation medium, and cell lysates were analysed for COL1A1, COL2A1 and SOX9. bACT was used as a loading control. **(F)** Gene expression (mRNA) analysis of six NP markers: keratin 19 (*KRT19*), Carbonic anhydrase XII (*CA12*), cluster of differentiation 24 (*CD24*), Forkhead box F1 (*FoxF1*), paired box 1 (*Pax1*) and pleiotrophin (*PTN*) in cultured AF (white bars) and NP cell isolates (black bars) in tissue from three independent donors: D2 (P1), D4 (P5) and D5 (P5). Marker gene expression was normalized to bACT levels. NP data presented are relative to AF values (per patient). ^a^Indicated *P*-values were obtained by comparing AF and NP values combined for all three donors. Statistical significance was assessed by Student’s *t*-test. *P < 0.05; **P < 0.01. *P*-values for AF vs NP comparisons per donor are listed in Additional file 1: Table S1.

Isolated primary NP cells have been reported to display a rounded morphology and AF cells a comparatively more elongated morphology [25,26]. As immortalization procedures and isolation and expansion of cell clones require cells to be adherent, we adapted culture conditions to a monolayer system. Under these adherent growth conditions, primary NP and AF cells both showed a spread-out morphology at early passages (Figure 1A). To validate their NP and AF phenotypes (and hence their tissue origin), we analysed a number of independent primary NP cultures (donors 1 to 5; see Table 1) at different passages for protein and mRNA marker expression (Table 2) and compared them to complementary AF cultures (Figures 1B and 1D to 1F). Primary cultures from two independent donors, D1 (P0) and D2 (P1), showed more prominent *COL2A1* mRNA expression in NP cultures and expressed *COL1A1* equally in NP and AF cultures (Figure 1B). COL2A1 protein was detectable in one of two low-passage NP isolates, but not in primary AF cells (Figure 1B). COL1A1 protein was clearly detectable in all AF cultures, but was considerably lower in primary NP cells. These findings are consistent with reported differences between AF and NP cells [27].

Cell isolates D4 and D5 were expanded to P5 for comparative analysis and cloning (Figure 1C). The chondrocyte phenotype is known to be unstable *in vitro*[28,29]. We therefore tested the differentiation capacity of primary IVD cell cultures. AF and NP cell isolates (P5) from two independent donors showed low amounts of COL2A1 and comparable COL1A1 protein levels (Figure 1D). Dmed induced SOX9 protein expression in primary NP and AF cultures (Figure 1E). In addition, COL2A1 was induced in Dmed in NP as well as AF isolates. Importantly, COL2A1 induction was clearly more prominent in NP cultures (Figure 1E), whereas COL1A1 expression was higher in AF cells (Figure 1E).

NP marker mRNA analysis for *CA12*, *CD24*, *FOXF1*, *PAX1*, *PTN* and cytokeratin 19 (*KRT19*) clearly discriminated between AF and NP cultures in samples from three independent donors (Figure 1F). *KRT19* mRNA was higher in NP compared to AF cultures (4- to 70-fold), as were *CA12* (1.5- to 3-fold), *CD24* (2- to 2.5-fold), *FOXF1* (1.9- to 2.3-fold), *PAX1* (1.2- to 15-fold) and *PTN* (2- to 3-fold). These six markers initially appeared stable in pools P0 to P5, although expression diminished thereafter, at P10 (data not shown). Of note, neither *T* mRNA nor protein was detected in the primary NP isolates of the donors, except for the youngest donor (D1, age 8 years) (Table 1). *T* is specifically expressed in the notochord and in NCs in the IVD. As such, this finding suggests that the primary isolates that we used for cell line generation were free of NCs. These combined phenotypic analyses confirmed the respective tissues of origin of the primary NP and AF cell isolates and suggest that differentiation capacity was maintained in primary NP cultures. Taken together, these findings provide a solid basis for experimental immortalization and clonal cell line generation.

**Figure 2:**
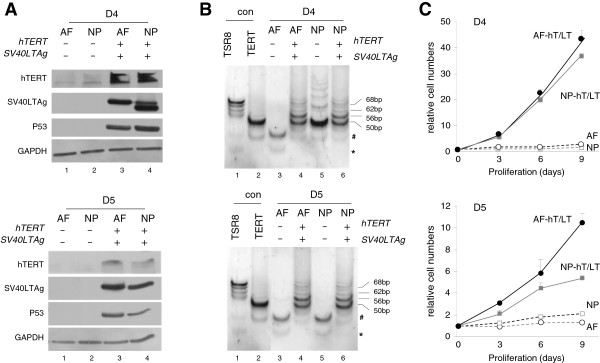
**Immortalization by retroviral transduction. (A)** Immunoblot analysis of human telomerase reverse transcriptase (hTERT), simian virus 40 large T antigen (SV40LTag) and P53 expression in cell lysates from mortal and immortal cells at extended culture periods (passage 20 (P20) for mortal and P40 for immortal cells). Cells were immortalized at P5. Glyceraldehyde 3-phosphate dehydrogenase (GAPDH) was used as a loading control. **(B)** Telomeric repeat amplification protocol assay demonstrating telomerase activity in immortalized donor 4 (D4) and D5 annulus fibrosus (AF) and nucleosus pulposus (NP) cells (lanes 4 and 6, respectively) compared to control cultures (primary cultures (P5), lanes 3 and 5). Lanes 1 and 2 represent positive controls for PCR amplification (TSR8 oligonucleotide synthesis reagent in the TRAPeze kit) and TERT, respectively. Lane numbers are indicated below the images. Fragment size is indicated in base pairs (bp). #Internal PCR standard. *Aspecific PCR products. **(C)** Spectrometric quantification of Crystal violet staining assays reflecting relative proliferation of immortalized and primary cells at each indicated time point (compared to baseline (*t* = 0)). P20 cultured primary cells had a negligible proliferative index as they approached 50 population doublings (PDLs). The immortalized cells had undergone approximately 160 PDLs (corresponding to ±40 passages) postimmortalization at this time point. hT/LT denotes cells transduced with hTERT (hT) and SV40LTag (LT).

Isolates from two different donors (D4 and D5) (Table 1) were immortalized at P5 using a combination of retroviral vectors expressing SV40LTag or hTERT. SV40LTag and hTERT expression and/or function were analysed in transduced AF and NP pools (Figures 2A and 2B). Assessment of the proliferative capacity of transduced AF and NP pools revealed that proliferation lifespan was extended well beyond that of the nonimmortalized parental cell pool (Figure 2C). Transduced, G418-resistant AF and NP cells were seeded at low density to allow expansion of single immortal colonies. A more detailed description of the procedure is provided in the Methods section. A detailed characterization of immortal AF clones will be published elsewhere.

**Figure 3:**
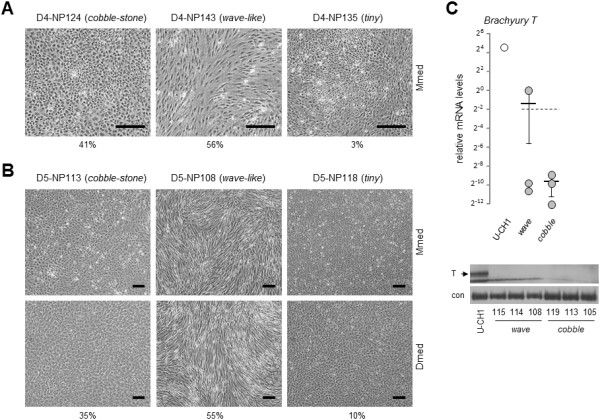
**Clonal phenotype of nucleus pulposus cell lines. (A)** Representative phase-contrast images of cobblestone, wavelike and tiny cell morphology of immortalized donor 4 (D4) nucleus pulposus (NP) cells. **(B)** Representative phase-contrast images of cobblestone, wave-like and tiny cell morphology of immortalized D5 NP cells. Images were captured upon confluence in maintenance or differentiation medium. The percentage of the total amount of clones per phenotype is indicated below the images. Black bars in = 20 μm. **(C)** Scatterplot (top) of relative *Brachyury T* expression levels in immortalized NP clones. The horizontal bar represents the average *T* mRNA level of all six NP clones represented in the immunoblot images (bottom), which show *T* protein levels in NP clones. Each data point represents a triplicate measurement for an individual clone. The solid black bars represent the averages associated with specific clonal morphology. The dashed bar represents the average of six clones combined. The U-CH1 cell line was used as a positive reference for both mRNA and protein expression analyses.

Immortal NP clones from each donor exhibited a set of distinct morphologies. During monolayer expansion, 40% (D4) and 35% (D5) of the clones showed a ‘cobblestone’ appearance and 56% (D4) and 55% (D5) were organized in wavelike patterns (Figures 3A and 3B and Table 1). A third, rare clonal phenotype consisted of distinctly smaller cells, denoted as ‘tiny (3% of D4 clones and 10% of D5 clones) (Figures 3A and 3B). Stimulation with Dmed further emphasized the differences in morphology (Figure 3B, bottom panel). Very low *T* mRNA expression (on average 100-fold lower than the U-CH1 positive control), and absence of T protein indicated that representative clones expressed few if any notochord markers (Figure 3C). The observation that these phenotypic subtypes could be established from two independent donor NP pools strongly suggests that multiple different cell phenotypes coexist in the human NP and that these can be isolated as NP subtypes and propagated as immortal cell lines.

### Phenotypic characterization of immortalized nucleus pulposus clones

We next examined the differentiation capacity of 20 individual immortalized D5 NP clones (Table 1). Expanded clones were induced to differentiate using a standard AC protocol with Dmed, and marker expression was evaluated at the level of proteins (SOX9, COL2A1 and COL1A1).

**Figure 4:**
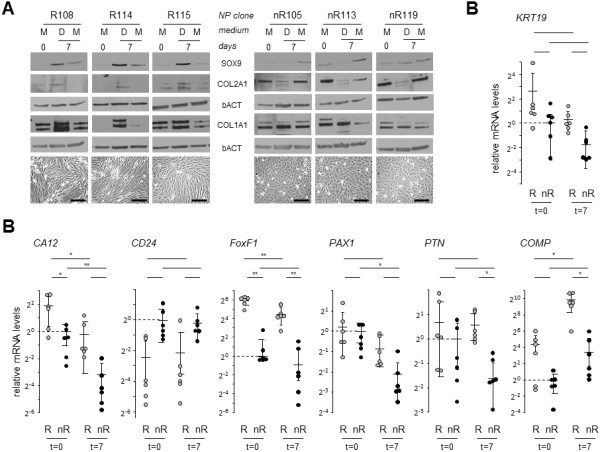
**Marker expression in clonal subtypes. (A)** Immunoblot analyses of collagen type II, α1 (COL2A1), SOX9 and collagen type II, α1 (COL1A1), in responder nucleus pulposus (NP-R) clones (left panels) and nonresponder nucleus pulposus (NP-nR) clones (right panels). Cell clones were incubated for 7 days with differentiation medium (Dmed; ‘D’ in figure) or maintenance medium (Mmed; ‘M’ in figure). The different subtype samples were run on the same gels to enable direct quantitative comparison. Three representative clones per NP subtype are shown. β-actin (bACT) was used as a loading control. Representative phase-contrast images of NP-R clones 108, 114 and 115 and of NP-nR clones 105, 113 and 119 are shown in the bottom row. Black bars = 20 μm. **(B)** (top right panel; continued in lower panels) Scatterplots of relative gene expression analysis of keratin 19 *KRT19*, Carbonic anhydrase XII (*CA12*), cluster of differentiation 24 (*CD24*), Forkhead box F1 (*FOXF1*), paired box 1 (*PAX1*), pleiotrophin (*PTN*) and cartilage oligomeric matrix protein (*COMP*) in six representative NP-R clones (104, 108, 114, 115, 121 and 124; grey symbols) and six representative NP-nR clones (102, 105, 110, 113, 116 and 119; black symbols) at baseline (*t*0) (Mmed) or at 7 days of culture in Dmed. Gene expression was normalized to bACT mRNA levels. Log_2_-scaled expression data are presented relative to the average expression level of NP-nR at *t*0. Each data point represents a triplicate measurement for an individual clone. The *P*-values were obtained by comparing the indicated experimental groups. Statistical significance was assessed by Student’s *t*-test. *P < 0.05; **P < 0.01. Full list of *P*-values is available in Additional file 1: Table S2.

The majority (90%) of clones displayed one of two responses based on their ability to undergo chondrogenic differentiation: responder NP (NP-R) clones showed a clear differentiation-dependent induction of SOX9 expression, whereas nonresponder (NP-nR) clones did not (Figure 4A). In NP-R clones, SOX9 induction correlated with COL2A1 induction, whereas this was not the case for NP-nR clones, which expressed higher basal COL2A1 levels at *t*0) than NP-R clones. COL1A1 protein was detectable in most clones in Mmed and relatively increased in NP-R clones in Dmed compared to NP-nR clones. Of note, the induction profiles correlated perfectly with clonal morphology: All chondrogenic NP-R clones exhibited the wavelike morphology, whereas all NP-nR clones displayed a cobblestone appearance (Figure 4A, lower panels). As the ‘tiny’ clone morphology did not show a consistent marker expression signature, it was omitted from further analysis.

To determine whether any of the NP markers described above could discriminate between the clonal subtypes (NP-R vs nR), we evaluated differences in mRNA levels of *KRT19*, *CA12*, *CD24*, *PTN*, *FOXF1* and *PAX1*. In addition, we included cartilage oligomeric matrix protein (*COMP*), a gene previously shown to be expressed in rat AC and NP cells and in human NP cells [30,31]. Comparative gene expression analysis of two groups of six representative chondrogenic NP-R and NP-nR clones revealed a clear segregation between the two subtypes. Of these markers, *CA12* and *FOXF1* levels were significantly higher at *t*0 in NP-R clones (Figure 4B; see Additional file 1: Table S2). *CD24* showed a trend towards lower expression in NP-R clones under maintenance conditions, whereas *COMP* mRNA levels tended to be higher in this NP subtype under the same conditions; however, these differences did not reach significance (*P* = 0.056 and *P* = 0.099, respectively). *KRT19*, *PAX1* and *PTN* mRNA levels were not significantly different between NP-R and nR clones at *t*0. Exposing NP subtypes to differentiation conditions induced distinct responses (that is, marker gene expression) between the NP subtypes. *CA12* mRNA levels significantly decreased in both NP-R and NP-nR clones, which eliminated the original differences (that is, at *t*0) between these subtypes (7 days in Dmed) (Figure 4B). In contrast, *FOXF1* and *COMP* significantly changed in NP-R clones, but not in NP-nR clones, in Dmed. Conversely, PAX1 levels significantly dropped in NP-nR clones, but not in NP-R clones, in Dmed. Remarkably, all seven markers tested displayed enhanced differences in expression levels in NP-R versus NP-nR clones under Dmed conditions, three of which reached significance (*FOXF1* (*P* = 0.008), *PTN* (*P* = 0.01) and *COMP* (*P* = 0.02)). The remaining four markers showed consistent trends (*CA12* (*P* = 0.069), *CD24* (*P* = 0.073), *PAX1* (*P* = 0.066) and *KRT19* (*P* = 0.056)) (Figure 4B). CD24 excepted, expression of all markers was reduced in NP-nR clones under these conditions. The induction of important ECM structural protein-encoding mRNAs such as *COL2A1* and *ACAN* was relatively low at the transcriptional level and did not correlate with cell morphology (data not shown).

In summary, the results of our analyses demonstrate clear differentiation-induced COL2A1 and SOX9 protein detection in NP-R clones that correlate well with the described morphological characteristics. The phenotypic differences between NP-R and NP-nR clones are further substantiated by differential NP marker gene expression profiles, under both nondifferentiation and differentiation conditions, and suggest functional phenotypic differences between the immortalized clonal subtypes.

### Cell surface characterization of nucleus pulposus clones

**Figure 5:**
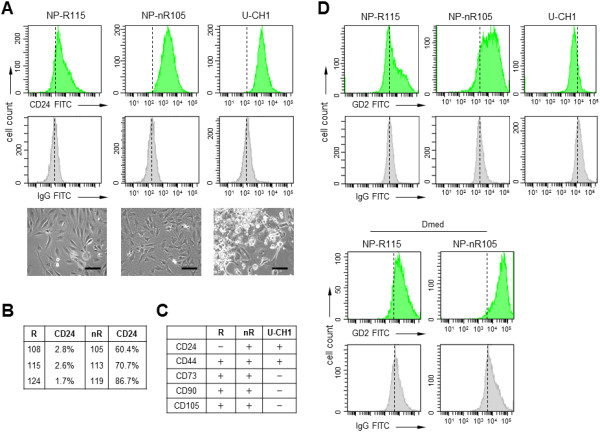
**Cell surface characterization of nucleus pulposus clones. (A)** Cluster of differentiation 24 (CD24) histograms (top panels; green) of representative responsive and nonresponsive nucleosus pulposus (NP-R and NP-nR, respectively) subtypes (NP-R115 and NP-nR105) and an immunoglobulin G (IgG)–negative control (middle panels; grey). Phase-contrast images are shown in the lower panels. FITC, Fluorescein isothiocyanate. Black bars = 20 μm. **(B)** Percentage of CD24^+^ cells in three independent NP-R and NP-nR clones. **(C)** CD marker scores for the indicated cell lines: NP marker CD24, the hyaluronic acid receptor CD44 and the mesenchymal stem cell markers CD73, CD90 and CD105. **(D)** Gangliosidase 2 (GD2) histograms of subconfluent cultures of representative NP subtypes in maintenance medium (Mmed): NP-R115 and NP-nR105 (top panels) and 7-day differentiated cultures in differentiation medium (Dmed) (bottom panels). The U-CH1 chordoma cell line was used as a reference.

On the basis of the differential marker expression profiles, we hypothesized that NP-R clones represent a NP stem cell progenitor-like phenotype, whereas the NP-nR clones might have been immortalized at a more advanced differentiation or maturation stage. To obtain further support for this supposition, we analysed a number of mesenchymal stem cell (MSC) surface markers (CD73, CD90 and CD105), a disc progenitor marker (GD2) and ontogeny markers (cell adhesion glycoprotein CD24, chondrocytic hyaluronan and collagen receptor CD44) [32-36]. We selected a number of NP-R and NP-nR clones for flow cytometry analysis based on (1) highest SOX9 and COL2A1 protein expression and (2) fold difference in basal *KRT19*, *FOXF1* and *CA12* gene expression levels (see Figure 4). We used the cell line U-CH1 was used as a reference [15]. In keeping with the *CD24* mRNA measurements, CD24 surface expression was most prominent in NP-nR clones and suggested a more advanced maturation stage (Figures 5A and 5B) [10]. Both NP subtypes were CD44^+^ (Figure 5C). In good correlation with their presumed more advanced differentiation stage, NP-nR clones showed more prominent GD2 positivity than NP-R clones under both basal conditions and differentiation conditions Figure 5D) [10]. Both NP clonal subtypes were CD90^+^/CD105^+^/CD73^+^ for these markers (Figure 5C). The observation that all cells were positive for one of the markers in a marker cocktail (CD14, CD20, CD34 and CD45) (data not shown) supports the argument that neither clonal subtype displayed pure MSC properties. Combined, however, the CD marker expression profiles of the NP subtypes do support a mesenchymal origin of the immortal NP clones. Moreover, the CD24/GD2 status discriminates between the NP-R and NP-nR subclones and suggests a difference in differentiation or maturation stage.

### Extracellular matrix molecules in nucleus pulposus cell differentiation

**Figure 6:**
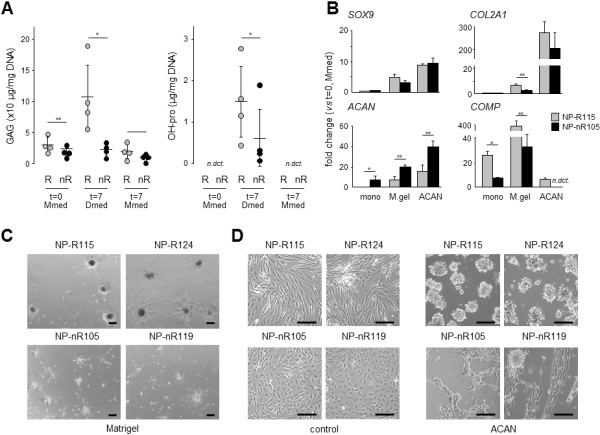
**Extracellular matrix molecules in nucleus pulposus differentiation. (A)** Extracellular matrix (ECM) properties in responsive nucleus pulposus (NP-R) clones (left column; R) and nonresponsive nucleus pulposus (NP-nR) clones (right columns; nR). Sulphated glucosaminoglycan (GAG; left graph) and hydroxyproline (right graph; OH-pro) quantities in four representative NP-R clones (108, 114, 115 and 124; grey circles) and four NP-nR clones (102, 105, 113 and 119; black circles) at baseline (*t* = 0), at 7 days in differentiation medium (Dmed) and at 7 days in maintenance medium (Mmed). Statistical significance was assessed by Student’s *t*-test. *P < 0.05; **P < 0.01; *n.dct*., Not detectable. Full list of *P*-values is provided in Additional file 1: Table S3. **(B)** Comparison of marker expression on monolayer (mono; uncoated polystyrene), Matrigel-coated (M.gel) and aggrecan- coated (ACAN) cultured NP cell clones. Gene expression levels of SRY-box 9 (*SOX9*), *COL2A1*, *ACAN* and cartilage oligomeric matrix protein (*COMP*) are depicted. Grey bars correspond to NP-R115, and black bars to clone NP-nR105. Gene expression was normalized to *β-ACTIN* mRNA levels. Data are expressed as fold changes relative to expression levels at baseline (*t* = 0) (Mmed). Statistical significance was assessed by Student’s *t*-test. Full list of *P*-values is provided in Additional file 1: Table S4. *P < 0.05; **P < 0.01; *n.dct.* = not detectable. **(C)** Phase-contrast images of representative NP-R clones and NP-nR clones on Matrigel-coated plates at 36 hours postplating. **(D)** Phase-contrast images of representative NP-R clones and NP-nR clones on monolayer plastic or ACAN coating at 72 hours postplating. Black bars = 20 μm.

The ECM provides a biological niche that plays a significant role in cellular responses and, as such, in tissue development, homeostasis and repair [37]. For this reason, we measured GAG synthesis and the formation of OH-pro groups in two sets of four independent clones, with each set representing a distinct NP subtype. As neither the exact culture methods nor the optimal composition of Dmed have been established for NP cells, we compared GAG and OH-pro formation in monolayer cultures. In addition, this allowed comparison with data collected to date for the current and other cell lines [14]. We found that, compared to NP-nR clones, NP-R clones produced more (fourfold) GAGs in response to Dmed (Figure 6A). In contrast, culture in Mmed for the same amount of time did not induce GAG production in either subtype (Figure 6A). Although the formation of OH-pro was induced in both NP clonal subtypes in response to Dmed, OH-pro levels reached significantly higher values in NP-R than in NP-nR clones (Figure 6A); no OH-pro was detectable at baseline or in Mmed. Thus, consistent with their distinctive responses to differentiation conditions (for example, induction of *SOX9* and *COL2A1*) (see Figure 4), NP-R clones produced more GAGs and OH-pro than NP-nR.

We next tested the differentiation capacity of NP-R and NP-nR cells on Matrigel, as this was recently shown to support differentiation of induced pluripotent stem cells (iPSCs) to NP-like cells [38]. In addition, we examined the effect of ACAN coating on NP subtype differentiation. ACAN is an important constituent of the NP ECM, and ACAN coating has been applied to assist differentiation of meniscus-derived fibrochondrocytes and MSCs into chondrocyte-like cells [39,40]. We cultured NP-R and nR cells in the presence of either Matrigel or ACAN for 3 to 7 days under differentiation conditions and compared induction of *SOX9*, *COL2A1*, *ACAN* and *COMP* expression. *COL1A1* expression was low under all conditions and was not induced (data not shown). *SOX9* mRNA levels were comparable between the NP subtypes under all conditions tested. Interestingly, *COMP* induction was consistently higher in NP-R cells, whereas, conversely, *ACAN* induction was consistently higher in NP-nR cells, providing further support for the maintenance of distinct functional properties between the NP-R and NP-nR clones. Only on Matrigel was *COL2A1* induced significantly greater in NP-R cells (Figure 6B). Remarkably, Matrigel and ACAN coating had dramatic effects on differentiation-induced *COMP* and *COL2A1* expression levels, respectively. These combined results support the idea that ECM is a determining factor in NP cell differentiation responses and suggest that distinct NP cell types differentially respond to the presence of ECM molecules.

The variation in differentiation capacity between NP-R and NP-nR clones suggested a possible difference in differentiation status or maturity between NP cellular subtypes. As relatively immature porcine NC and NP cells were shown to form spheroids on Matrigel [41], we compared NP-R and NP-nR clones for their ability to form spheroids structures under similar conditions. NP-R clones produced spheroid structures on Matrigel, whereas growth of NP-nR clones appeared to be restricted to small adherent colonies (Figure 6C). Remarkably, on ACAN, the NP subtypes revealed similar responses: NP-R clones showed poor attachment and readily formed floating spheroids (Figure 6D). Spheroid formation occurred within 24 hours and was most prominent at 72 hours postplating. In contrast, NP-nR clones maintained surface adherence, and spheroid formation (that is, adherence) appeared only sporadically (see, for example, Figure 6D). These collective findings suggest that distinct biological responses are triggered in NP subtypes in the presence of ECM and that Matrigel and ACAN evoke gene-specific transcriptional responses in NP cells compared to standard monolayer differentiation assay conditions.

## Discussion

The application of human IVD material as a potential source of cell lines to date has been limited. Sakai *et al.* published their first immortalized HNPSV-1 cell line in 2004 [12]. The authors of a recent report [13] described the generation of an NP cell line utilizing a distinct immortalization strategy that we employed in our present study. Despite the low number of studies in which investigators have utilized immortalized NP cell lines, such models provide valuable tools with which to study important aspects, such as the developmental origins of NP, NP cell biology disc degeneration, and can also aid in drug development and drug testing. In this report, we present the generation and thorough initial characterization of novel clonal human NP cell lines. We have established multiple NP cell lines starting from immortalized primary (P5) NP cultures derived from two independent donors. Importantly, analysis of immortal NP clones shows that multiple clonal subtypes can be distinguished based on morphological characteristics, marker gene expression and their diverse responses to differentiation conditions. We also report that ECM-assisted culture models further segregate the NP-R and nR phenotypes based on their spheroid-forming capacity in culture.

### Nucleus pulposus marker expression

Several reported NP markers expressed by our immortal cell clones are consistent with a human NP phenotype and hence distinguish our primary NP and AF cultures. NP cell cultures typically synthesize COL2A1, ACAN and SOX9, whereas AF cells predominantly produce COL1A1 and considerably less COL2A1 [42]. Cytokeratin 8 (CK8) and cytokeratin 19 (KRT19) have been suggested as potential NP markers [5,33,43]. Indeed, our NP primary cultures clearly segregated from AF cultures based on *KRT19* mRNA expression. In line with the report of cell surface marker CD24 being able to identify the immature NP [44], we found CD24 expression in primary NP cells. Although none of the markers used in the present or previously published analyses are exclusively linked to AF, NP or AC tissue, the consistency of the quantitative differences we detected between AF- and NP-derived cultures further substantiates correspondence to their tissue of origin.

We report here, for the first time to the best of our knowledge, that the significant differences in marker expression between immortal NP subtypes isolated from immortalized primary cultures (*CA12*, *CD24*, *FOXF1*, *PTN*, *PAX1* and *COMP*) discriminate human NP subtypes from two independent donors in primary monolayer cultures. Relevantly, the *CA12*, *CD24*, *FOXF1* and *KRT19* genes have been used successfully to study IVD-like differentiation of MSCs [45]. Of note, throughout the present study, we used differentiation conditions that have previously been established for AC models. As the most optimal conditions for NP cell differentiation are currently unknown, some caution is warranted in direct translation of this data to the *in vivo* situation. Likewise, numerous animal studies have revealed NP markers that either fail as human NP markers or have not been evaluated in human IVD tissue. Thus, despite recent advances, a consistent definition of a NP marker signature for human cells *in vivo* is currently lacking [7,46]. Investigators in recent gene expression array studies have identified additional markers that distinguish human NP cells from AC and/or AF cells [3,5]. Similarly, gene expression analysis of distinctive immortalized cellular subtypes may prove useful in refinement of NP marker definition and is also expected to contribute to the development of robust differentiation protocols. Clearly, additional functional analyses are required to determine the role of NP markers in the development and pathology of the IVD.

### Nucleus pulposus cell models and differentiation protocols

Using primary tissue from scoliosis patients as starting material justifies the question whether the primary culture and the immortalized cell cultures may contain degenerate cells. Although we cannot formally exclude this possibility based on our marker analysis, the IVD material we used for these studies was mainly obtained from the convex (that is, noncompressed) side of scoliotic discs. Of note, all available donors were relatively young. In addition, although the number of studies on DDD in the context of scoliosis are limited, DDD is rarely detected at this age, with the exception of cases with associated morbidity (for example, concurrent congenital spinal stenosis, body mass index >30, athletics) [47].

It is important to note that the application of immortal cell lines is restricted to *in vitro* analyses. Experimental application may be limited as a direct consequence of the immortalization procedure itself. For example, SV40LTag interferes with pRB and TP53 function; both proteins are involved in the programming of senescence, which may be relevant for the degeneration process [48-52]. However, the establishment of distinct NP clonal phenotypes reported herein (for the first time, to the best of our knowledge) provides access to a source of human NP cell models that will enable us to exploit differences in aspects such as cell surface marker expression to study NP cell biology. These immortalized NP cell models should thus be viewed as powerful complementary models to primary isolates.

The identification of distinct cell phenotypes among our immortalized NP cell clones reflects the cellular heterogeneity *in vivo* and provides important avenues for further study. Our observations reported herein support the notion that it is possible to fix different epigenetic states (that is, functional cellular heterogeneity) in the NP by means of immortalization procedures. Indeed, others have observed that cellular phenotypes are retained by immortalization [53]. It is relevant to note that NP-R and NP-nR clonal outgrowth may have been subject to selection bias, as integration of retroviral DNA is dependent on active *de novo* DNA replication; as such, the heterogeneity in NP tissue may not be limited to the two subtypes described herein.

Many differentiation protocols have been optimized for SOX9, COL2A1 or GAG expression in AC cultures, and such protocols have been used to differentiate bone marrow– or adipose tissue–derived MSCs towards an NP-like phenotype [54]. Also, TGFβ3 supplementation reproducibly promotes collagen and GAG synthesis in primary NP cells [31,55]; yet, the exact conditions that faithfully support NP differentiation (and appropriately functioning matrix formation) have been reported throughout the literature to vary and thus appear not to be fully optimized (a parameter that is dependent on consensus definitions of NP phenotypes). Our observation that TGFβ3 repressed expression of novel NP markers in primary cells and cell clones is consistent with a recent report [45]. Coculture experiments employing MSCs or conditioned media indicate that as yet unknown factors contribute to NP differentiation and/or phenotype stabilization [56-59]. The presence of Matrigel or ACAN produced a stronger induction of conventional chondrogenic markers in our clonal NP cell lines. Similarly, ACAN coating has been reported to induce a chondrocyte-like phenotype in primary fibroblasts, which upregulated *COL2A1* and *SOX9* but not *ACAN*, and primary meniscus fibrochondrocytes induced *ACAN* expression but not *COL2A1* and *SOX9*[19,39]. Also, bone marrow stromal cells express *ACAN* in response to ACAN coating culture [40]. Remarkably, we found that Matrigel and ACAN enhanced induction of *COMP* and *COL2A1* expression, respectively. Although the mechanism by which ACAN affects differentiation is currently unclear, it is possible that ACAN functions to enhance growth factor signalling, as was recently shown for COMP [60]. In this light, it would be of interest to determine the effect of ACAN coating on NP phenotype stability. Clearly, a much improved definition of NP maintenance and differentiation conditions is needed.

### Nucleus pulposus cellular subpopulations

Evidence for the existence of a progenitor population in the adult human disc *in vivo* is accumulating [3,10,61,62]. The immature NP contains NCs that harbour stem cell–like properties, and their lifelong presence in small laboratory animals, in contrast to humans, is thought to support tissue repair and thus prevent disc degeneration [63,64]. NCs are replaced by smaller, rounded, chondrocyte-like cells in early human adulthood [65]. As increasing evidence points to an ontogenic relationship between the notochord and the NP, [66], expression analysis of the NC marker *T* was of relevance to pinpointing the exact nature of our cell cultures. We found that T mRNA and T protein expression were very low and undetectable, respectively, in the primary isolates from which the immortal clones were derived. Only the primary NP isolate (D1P0) from the youngest donor (8 years old) had detectable T mRNA levels (Table 1). These data are consistent with reports that T expression disappears during human IVD maturation and is virtually undetectable from 10 years of age onwards [7,67]. In keeping with this, the low or absent T expression in our immortal clones suggests that not NCs, but rather more mature NP cells, were immortalized.

On the basis of their morphology and ability to induce *SOX9* and/or *COL2A1* expression, we hypothesize that our different immortalized subtypes may represent more differentiated (NP-nR, cobblestone) or more progenitor-like (NP-R, wavelike) cell types. Importantly, we found both subtypes in two independent patient isolates (one male, one female). Both subtypes were CD44^+^, CD73^+^, CD90^+^ and CD105^+^, consistent with previously published data [7]. CD24 and GD2 appeared to be more prominently present in our NP-nR clones than in NP-R clones, again supporting NP cell heterogeneity. Future characterization is needed to ascertain the ontogeny of the cloned cells.

The enhanced capacity to form spheroids under Matrigel and ACAN-supported culture conditions favours the notion that the NP-R cells represent a more progenitor or immature cell. Spheroid formation in culture is believed to mirror stem and progenitor capacity in any cell population. Lung-derived bronchospheres (or neurospheres, pancreatic) were previously shown to have stem cell– and progenitor-like characteristics [68,69]. Indeed, spheroid formation was also recently used to identify progenitor cell presence in the IVD [10]. The NP-R and NP-nR clones classify as GD2^−^/CD24^−^ and GD2^+^/CD24^+^, respectively, and the spheroid-forming capacity of a subset of primary NP cells was recently used to propose a differentiation map for Tie2, GD2 and CD24 [10]. Whether the phenotypes (chondrocyte-like, spheroid-forming and/or repopulating cells vs fibroblast-like cells) match our NP-R and NP-nR phenotypes and what role they fulfil in human NP development and disease remain to be determined. At this point, it is also premature to speculate about the ontogeny of these cells.

## Conclusions

We report, for the first time to the best of our knowledge, the generation of phenotypically distinct immortal subclones from primary human NP tissue. Characterization of these clones suggests they correspond to discrete subpopulations in the NP, which differ in terms of morphology, cell surface NP marker expression and differentiation capacity. Interestingly, AC cell lines generated in the 20th century continue to provide important new insights into fundamental issues relating to AC cell biology [70]; thus, similarly immortal NP cell lines are expected to provide vital tools with which to study molecular mechanisms of NP cell ontogeny and cell function in the healthy and diseased IVD. It has become clear that the NP chondrocyte differs from AC; yet, there is currently no comprehensive, descriptive consensus regarding the NP phenotype. We advocate a systematic analytical coverage of isolation procedures, culture conditions and NP marker expression. The environmental interplay between chondrogenic factors and ECM proteins provides clues for further research. How to define the NP cell and how to faithfully mimic the NP microenvironment *in vitro* remain crucial questions to be resolved in the IVD field. Such issues will determine the success of cell replacement therapy and/or tissue engineering approaches to treating DDD.

## Abbreviations

AC: Articular cartilage; ACAN: Aggrecan; AF: Annulus fibrosus; bACT: Β-actin; CA12: Carbonic anhydrase XII; CD24: Cluster of differentiation 24; COL1A1: Collagen type I, α1; COL2A1: Collagen type II, α1; COMP: Cartilage oligomeric matrix protein; DDD: Degenerative disc disease; Dmed: Differentiation medium; ECM: Extracellular matrix; FOXF1: Forkhead box F1; GAG: Glucosaminoglycan; GD2: Gangliosidase 2; hTERT: Human telomerase reverse transcriptase; iPSC: Induced pluripotent stem cell; IVD: Intervertebral disc; KRT19: Keratin 19; Mmed: Maintenance medium; NC: Notochordal (precursor) cell; NP: Nucleus pulposus; NP-nR: Nonresponder nucleus pulposus; NP-R: Responder nucleosus pulposus; OH-pro: Hydroxyproline; PAX1: Paired box 1; pRB: Retinoblastoma protein; P: Passage number; PTN: Pleiotrophin; RT-qPCR: Real-time quantitative polymerase chain reaction; SOX9: SRY-box 9; SV40LTag: Simian virus 40 large T antigen; T: Brachyury T; Tie2: Angiopoietin-1, tyrosine kinase receptor 2; TP53: Transforming protein 53.

## Competing interests

This research forms part of BMM-IDiDAS P2.01 project (‘Biomaterials for Diseases of the Intervertebral Disc’, a research programme of the BioMedical Materials Institute), which is cofunded by the Dutch Ministry of Economic Affairs, Agriculture and Innovation.

## Authors’ contributions

GGHA was responsible for study design; performed all tissue sample preparation, immortalization, molecular studies data analysis; and drafted the manuscript. DAMS, AC, RRP and SMR helped in study coordination, analysis and interpretation and presentation of the data. JAH, LR, TJMW and JWV conceived the study, secured funding, participated in study design and coordination, analysed the results and cowrote the manuscript. All authors read and approved the final manuscript.

## Supplementary Material

Additional file 1: Table S1 Statistical analyses for **Figure** 1**F.Table S2.** Statistical analyses for **Figure** 4**B.Table S3.** Statistical analyses for **Figure** 6**A.Table S4.** Statistical analyses for **Figure** 6**B.** Click here for file
