# Supplementary material for: Novel immortal human cell lines reveal subpopulations in the nucleus pulposus
Source: Arthritis Res Ther. 2014 Jun 27;16(3):R135. doi: 10.1186/ar4597 (PMC4227062; doi:10.1186/ar4597)
Supplement: Additional file 1: Table S1 — Statistical analyses for Figure 1F.Table S2. Statistical analyses for Figure 4B.Table S3. Statistical analyses for Figure 6A.Table S4. Statistical analyses for Figure 6B. [file ar4597-S1.pdf]

Additional file 1

marker	Donor 2	Donor 4	Donor 5
CA12	**0.0001**	**0.017**	0.077
CD24	**0.04**	**0.03**	0.07
FoxF1	0.08	0.17	**0.03**
PAX1	0.370	**0.002**	**0.009**
PTN	0.09	0.07	0.06
KRT19	**0.03**	**0.01**	**0.02**

Table S1. Statistical analyses for Figure 1F

AF and NP cultures were compared and p-value is reported per donor for each gene. Student's *t*-test was used to assess significance. P-values are reported for each marker; statistically significant differences are printed in bold.

marker	t0<br>R vs nR	t7<br>R vs nR	R<br>t0 vs t7	nR<br>t0 vs t7
KRT19	0.2926	0.0555	0.2644	0.0730
CA12	**0.0352**	0.0694	**0.0430**	**0.0126**
CD24	0.0559	0.0729	0.7170	0.4545
FoxF1	**0.0004**	**0.0075**	**0.0008**	0.1766
PAX1	0.4150	0.0658	0.1549	**0.0158**
PTN	0.2108	**0.0102**	0.7604	0.1986
COMP	0.1000	**0.0208**	**0.0171**	0.1535

Table S2. Statistical analyses for Figure 4B

6 representative clones for each NP subtype were cultured in Mmed or in Dmed. Average expression values between clonal subtypes (R vs nR) were statistically compared at t=0 (Mmed) or at t=7 (Dmed), or between t=0 and t=7 (t0 vs t7), for each subtype to the corresponding t=0 condition. Student's *t*-test was used to assess significance. P-values are reported for each marker; statistically significant differences are printed in bold.

GAG	R vs nR	OH-pro	R vs nR
Mmed t0	**0.012**	Mmed t0	*n.dtc*
Dmed t7	**0.044**	Dmed t7	**0.022**
Mmed t7	0.160	Mmed t7	*n.dtc*

Table S3. Statistical analyses for figure 6A

6 clones per NP subtype were analysed for GAG content or OH-pro content. Averages of 6 clones were statistically compared between clonal subtype (R vs nR) at t=0 (Mmed), t=7 (Dmed) or at t=7 (Mmed). Student's *t*-test was used to assess significance. P-values are reported for each marker, statistically significant differences are printed in bold; *n.dtc.* indicates that levels were too low to detect.

	NP-R vs nR		
marker	monolayer	Matrigel	ACAN
SOX9	0.09	0.21	0.52
COL2A1	0.71	**0.01**	0.50
ACAN	**0.05**	**0.01**	**0.01**
COMP	**0.01**	**0.01**	*n.dtc*

Table S4. **Statistical analyses for figure 6B**

Fold inductions of indicated genes was compared per clone for monolayer, Matrigel or Aggrecan coated differentiation assays. The value's for t=0 were set to 1 for each culture method. Student's *t*-test was used to assess significance. P-values are reported for each marker; statistically significant differences are printed in bold.
